# Growing impact of wildfire on western US water supply

**DOI:** 10.1073/pnas.2114069119

**Published:** 2022-02-22

**Authors:** A. Park Williams, Ben Livneh, Karen A. McKinnon, Winslow D. Hansen, Justin S. Mankin, Benjamin I. Cook, Jason E. Smerdon, Arianna M. Varuolo-Clarke, Nels R. Bjarke, Caroline S. Juang, Dennis P. Lettenmaier

**Affiliations:** ^a^Department of Geography, University of California, Los Angeles, CA 90095;; ^b^Lamont-Doherty Earth Observatory of Columbia University, Palisades, NY 10096;; ^c^Cooperative Institute for Research in Environmental Sciences, University of Colorado Boulder, Boulder, CO 80309;; ^d^Civil, Environmental and Architectural Engineering, University of Colorado Boulder, Boulder, CO 80309;; ^e^Department of Statistics, University of California, Los Angeles, CA 90095;; ^f^Institute of the Environment and Sustainability, University of California, Los Angeles, CA 90095;; ^g^Cary Institute of Ecosystem Studies, Millbrook, NY 12545;; ^h^Department of Geography, Dartmouth College, Hanover, NH 03755;; ^i^NASA Goddard Institute for Space Studies, New York, NY 10025;; ^j^Department of Earth and Environmental Sciences, Columbia University, New York, NY 10027

**Keywords:** wildfire, streamflow, climate change

## Abstract

Streamflow often increases after fire, but the persistence of this effect and its importance to present and future regional water resources are unclear. This paper addresses these knowledge gaps for the western United States (WUS), where annual forest fire area increased by more than 1,100% during 1984 to 2020. Among 72 forested basins across the WUS that burned between 1984 and 2019, the multibasin mean streamflow was significantly elevated by 0.19 SDs (*P* < 0.01) for an average of 6 water years postfire, compared to the range of results expected from climate alone. Significance is assessed by comparing prefire and postfire streamflow responses to climate and also to streamflow among 107 control basins that experienced little to no wildfire during the study period. The streamflow response scales with fire extent: among the 29 basins where >20% of forest area burned in a year, streamflow over the first 6 water years postfire increased by a multibasin average of 0.38 SDs, or 30%. Postfire streamflow increases were significant in all four seasons. Historical fire–climate relationships combined with climate model projections suggest that 2021 to 2050 will see repeated years when climate is more fire-conducive than in 2020, the year currently holding the modern record for WUS forest area burned. These findings center on relatively small, minimally managed basins, but our results suggest that burned areas will grow enough over the next 3 decades to enhance streamflow at regional scales. Wildfire is an emerging driver of runoff change that will increasingly alter climate impacts on water supplies and runoff-related risks.

## Significance

How will increasing wildfire activity affect water resources in the water-limited western United States (WUS)? Among basins where >20% of forest burned, postfire streamflow is significantly enhanced by an average of approximately 30% for 6 y. Over 2015 to 2020, several large WUS basins experienced >10% of forest burned. Climate projections and an exponential forest fire response to climate-induced drying suggest the next 3 decades will see repeated years when WUS forest fire area exceeds that of 2020, which set a modern record for forest area burned. If so, entire regions will likely experience more streamflow than expected, potentially enhancing human access to water but posing hazard management challenges. Projections of water supply and runoff-related hazards must account for wildfire.

Recent declines in soil moisture, streamflow, and reservoir storage signal the precariousness of water supplies in the western United States (WUS) and the urgency of managing associated risks (1, 2). Declining WUS water supplies are qualitatively consistent with modeled trends due to anthropogenic climate change (3, 4), but projections are uncertain due to not only climate but also the complexity of vegetation responses to climate change and associated disturbances such as wildfire (5–9). In addition to transpiration and interception, which directly divert moisture from runoff, vegetation also affects hydrology by shaping soil depth and structure and by modulating turbulent energy fluxes that alter snowpack and evaporation (10). In addition to direct effects on vegetation, wildfires can further affect streamflow by promoting water repellency and soil erosion (11–13). Given that the headwater areas of major WUS rivers are generally forested, altered forest cover or ecosystem water demand could potentially affect water resources at regional scales.

In recent decades, the annual forest area burned in the WUS has risen rapidly, in step with climate trends toward warming and drying (14–20). In general, forest disturbances such as wildfire are known to temporarily enhance streamflow (21–23), although cases of postdisturbance streamflow declines, especially in arid areas, have also been documented (21, 24, 25). The likelihood that rapid increases in regional forest fire activity will continue (26, 27) suggests that wildfire may increasingly impact water resources in the water-limited WUS (6). Yet, the duration and seasonality of postdisturbance increases in runoff are unknown, raising the question of whether increased forest fire activity will meaningfully affect water availability in the WUS.

Here we use stream gauge records from 179 river basins in the WUS to assess the strength, duration, and seasonality of postfire changes in streamflow and whether increasing forest fire activity is likely to have a detectable effect on regional streamflow.

## Results

**Fig. 1.**
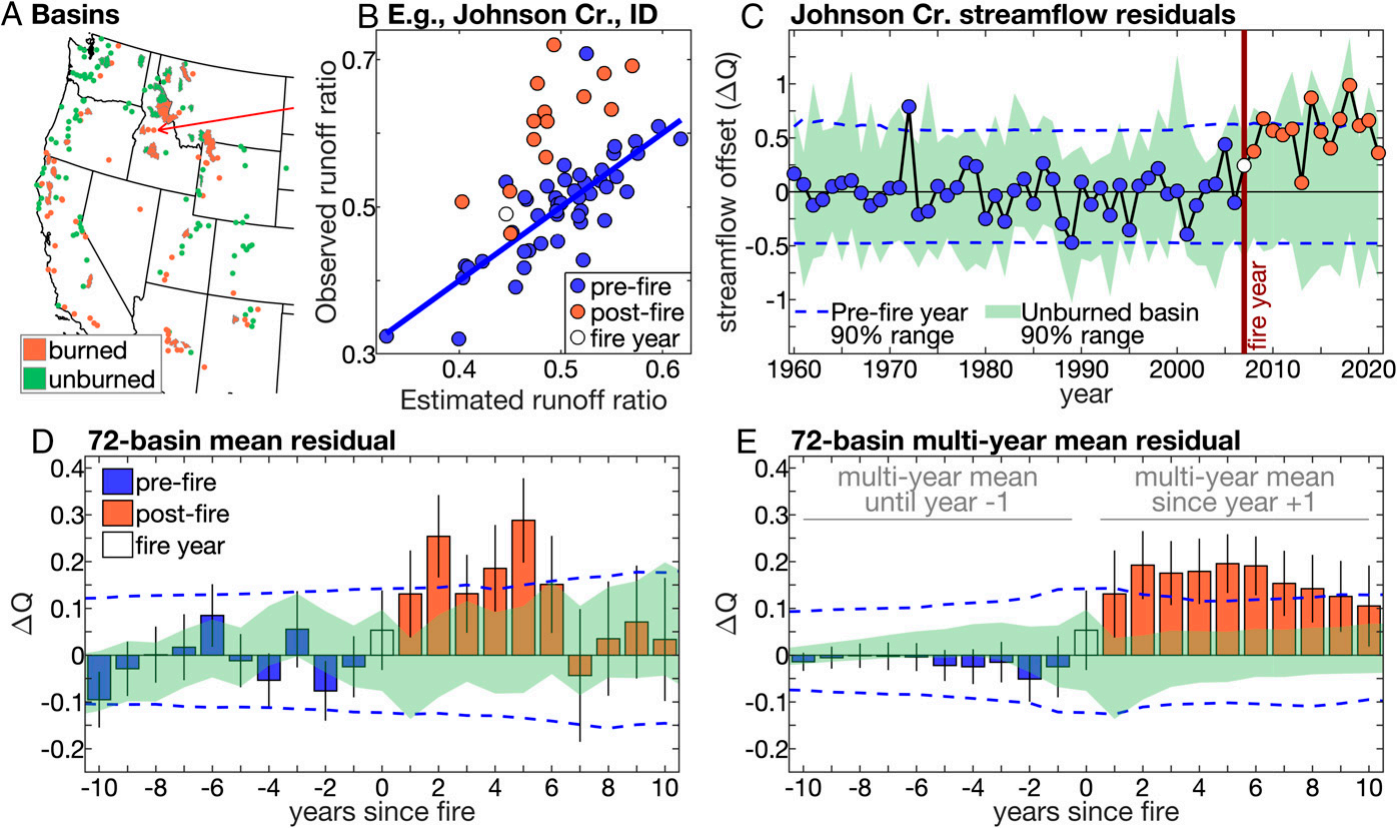
Effect of forest fire on water year streamflow. (*A*) Map of 72 burned (orange) and 107 unburned (green) basins (basins <1,000 km^2^ are indicated with a dot at the gauge location). Red arrow indicates Johnson Creek, ID (*B* and *C*). (*B*) Observed versus estimated water year runoff ratio for Johnson Creek, ID (blue line indicates prefire regression line). (*C*) Water year streamflow offset (ΔQ) at Johnson Creek. (*D*) Average ΔQ among burned basins in years prior to (blue bars), during (clear bar), and after (orange bars) each basin’s fire year. (*E*) Same as *D* but for multiyear means leading up to and following each basin’s fire year (e.g., year 6 is the mean of years 1 to 6 postfire). Black vertical lines indicate 90% bounds on means. Blue dashed lines indicate inner 90% when each basin’s ΔQ is replaced with 10,000 synthetic time series with prefire variance. Green area indicates inner 90% of 10,000 repetitions with unburned basins.

Among 179 minimally managed, forested basins in the WUS with long-term gauge records of streamflow, 72 experienced at least one large (>4.04 km^2^) wildfire during 1984 to 2019, and 107 experienced little to no wildfire during this time according to high-resolution satellite data (28) (Fig. 1*A* and see *SI Appendix*, Supplementary Text S1 for details on basin selection). To assess wildfire effects on streamflow, we calculate an annual streamflow offset (ΔQ) for each basin, which is a standardized time series of observed water year (October to September) streamflow (Q_obs_) minus that estimated from climate (Q_est_). To calculate Q_est_, we model water year runoff ratios for all available years during 1960 to 2021 in each basin based on prefire relationships between runoff ratios and climate and then multiply the modeled runoff ratios by the water year precipitation rate in the basin (*Materials and Methods*). For each burned basin, the fire year is the year when the greatest proportion of forest area in the basin was burned. Thus, ΔQ values are SD anomalies (σ) in Q_obs_ not accounted for by prefire climate–streamflow relationships. In the example basin represented in Fig. 1 *B* and *C*, ΔQ is positive in all 14 postfire years, meaning that postfire streamflow is higher than expected. Throughout the study, significance of ΔQ is assessed with two bootstrapping tests that characterize the expected range of variability in ΔQ without fire based on 1) records of ΔQ for the 107 unburned basins and 2) synthetic records of ΔQ representing each burned basin’s prefire period. Observed ΔQ must exceed 95% of bootstrapped values in both tests to be assessed as significantly positive (*Materials and Methods*).

Although single-year postfire ΔQ is often insignificant for individual basins, significant increases in postfire ΔQ emerge when multiple burned basins are assessed in aggregate (Fig. 1 *D* and *E*). Averaging across all burned basins, ΔQ is positive in each of the first 6 y postfire, significantly so in years 2, 4, and 5 (Fig. 1*D*). Runoff ratios are also significantly enhanced postfire, indicating that the postfire streamflow boost is not due to changes in precipitation (*SI Appendix*, Fig. S1). Beyond 6 y postfire, the all-basin means of ΔQ and runoff–ratio offsets drop abruptly.

Streamflow offsets can be quite variable among years and basins, but it is highly improbable for multibasin mean ΔQ to be positive for several consecutive years without wildfire. Averaging the 72-basin mean ΔQ across the first 6 y postfire yields a postfire streamflow offset of +0.19 σ, which is significant (*P* < 0.01) (Fig. 1*E*). Because postfire ΔQ is noisy among years and basins, all further analyses assess prefire and postfire ΔQ averaged over multiple years and basins, as is done in Fig. 1*E*. Fig. 1*E* extends to 10 y postfire to demonstrate that temporary streamflow offsets postfire can be relevant to water resources beyond the timescale of streamflow recovery.

**Fig. 2.**
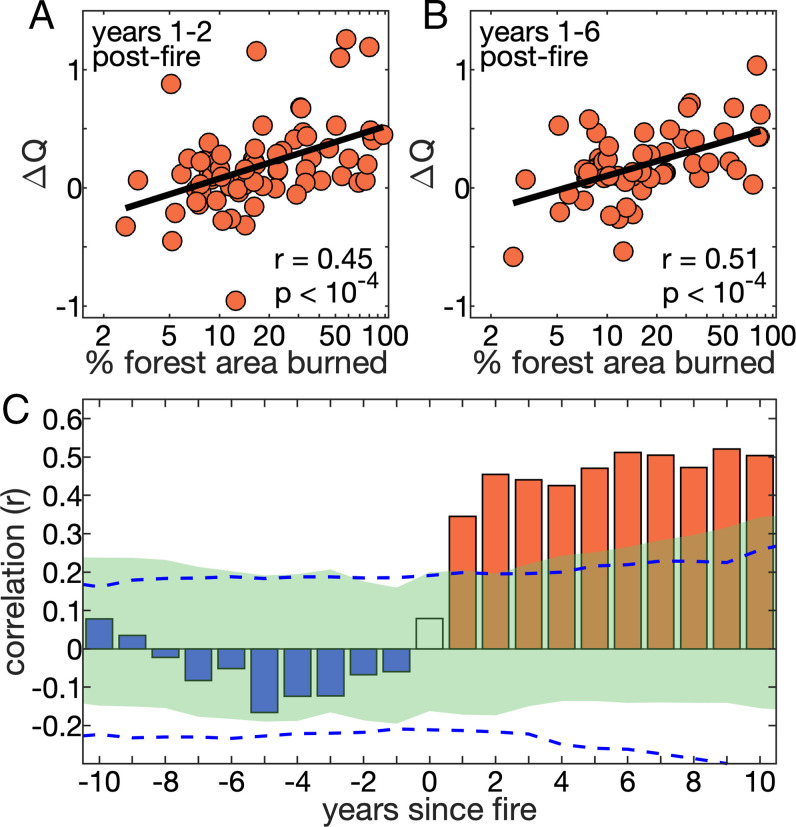
Proportion of forest area burned affects postfire runoff boost. Interbasin regression of the streamflow offset (ΔQ) averaged over the first (*A*) 2 and (*B*) 6 y postfire against the logarithm of the percentage of each basin’s forest area that burned in the fire year. (*C*) Interbasin correlation when repeating the analysis for other multiyear periods leading up to (blue bars) and after (orange bars) the fire year (clear bar). Blue dashed lines bound the inner 90% of correlation values when each basin’s ΔQ is replaced with 10,000 synthetic time series of ΔQ with prefire variance. Green area bounds the inner 90% of 10,000 repetitions with unburned basins. In *A* and *B*, years 1 to 2 and years 1 to 6 are shown because years 1 to 2 are when the all-basin mean streamflow enhancement is first significantly positive (Fig. 1*E*), and years 1 to 6 represent the full postfire period when all-basin mean streamflow was positive in all years (Fig. 1*D*).

Interbasin variability in postfire ΔQ is significantly (*P* < 10^−4^) and positively correlated with the percentage of forest area that burned in each basin (Fig. 2), supporting the causal link between forest fire and enhanced postfire streamflow. Positive postfire streamflow offsets are most common among basins where more than ∼20% of forest area burned (Fig. 2 *A* and *B*). Twenty percent has emerged repeatedly as a coarse estimate of a disturbance threshold associated with runoff increases (9, 10, 21, 22, 29, 30), but the high degree of scatter in Fig. 2 *A* and *B* indicates that postfire streamflow is affected by far more than the percentage of forest area burned, such as fire severity (9, 10, 22). Correlation is indeed marginally higher when we replace percent forest burned with satellite-derived estimates of percent of forest area burned by high-severity, stand-replacing wildfire from Parks and Abatzoglou (17). However, data were only available to assess stand replacement for only 64 of our 72 burned basins, and many basins experienced zero stand-replacing fire, strongly skewing the dataset (*SI Appendix*, Fig. S2). Furthermore, we might expect postfire streamflow increases to be largest not simply where a large fraction of forested area was burned but more specifically where a large fraction of the basin’s total area was burned in forest fire. However, the interbasin correlations discussed above are slightly weakened when the predictor variable represents the fraction of a basin’s total area burned in forest fire, stand-replacing forest fire, or wildfire regardless of vegetation type (*SI Appendix*, Fig. S3 *A*–*C*). Additionally, correlations are nearly eliminated when we consider wildfire in nonforested areas only, which are dominated by grasses and shrubs (*SI Appendix*, Fig. S3 *D* and *E*). Other factors that affect postfire runoff include climate, topography, and type of vegetation that succeeds postfire (10, 22, 31), but we find no relationship between postfire ΔQ and basin size, elevation, slope, aspect, mean annual precipitation, or fractional forest coverage.

**Fig. 3.**
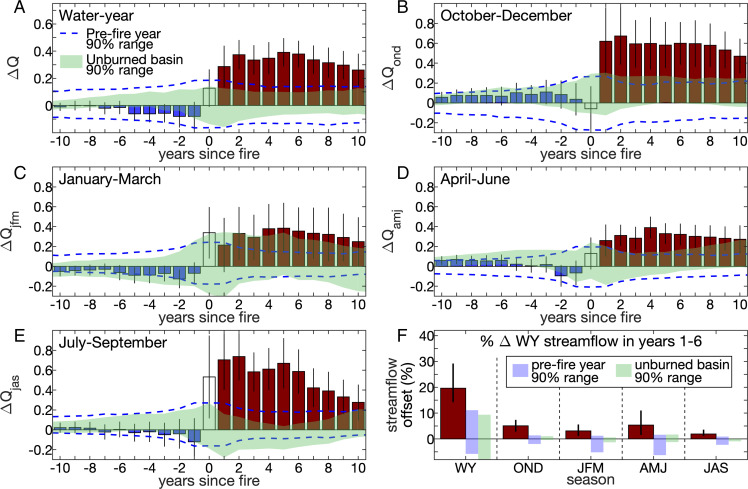
Multiyear mean water year and seasonal streamflow offsets in heavily burned basins. Streamflow offsets (ΔQ) averaged across only basins where >20% of forest area burned for (*A*) the water year and (*B*–*E*) the four seasons. Bars indicate multiyear means leading up to (blue) and after (dark red) the fire year (clear). Black vertical lines indicate 90% bounds on means. (*F*) Dark red bars indicate multibasin median streamflow offset averaged across years 1 to 6 postfire, expressed as percent of estimated total water year (WY) streamflow for the WY and each season: OND, JFM, AMJ, and JAS. Black vertical lines indicate 90% bounds on medians. Blue dashed lines in *A*–*E* and blue vertical areas in *F* bound the inner 90% when each basin’s ΔQ is replaced with 10,000 synthetic time series with prefire variance. Green background in *A*–*E* and green vertical areas in *F* bound the inner 90% of 10,000 repetitions with unburned basins.

Given that postfire ΔQ is largest among the more heavily burned basins, we now focus on the ∼40% of burned basins where >20% of forest area burned in the fire year. Among these heavily burned basins, the multibasin mean single-year ΔQ is significant (*P* < 0.05) in each of the first 6 y postfire and then remains positive but insignificant for years 7 to 10 (*SI Appendix*, Fig. S4). Averaged across the first 6 y postfire, mean ΔQ is +0.38 σ, exceeding the level achieved by any of our bootstrapped resamplings (*P* < 10^−4^) (Fig. 3*A*). Among individual basins, 38% of these basins experienced significant (*P* < 0.05) 6-y mean postfire ΔQ. Postfire mean ΔQ is also positive when averaged across basins where <20% of forest area burned but not significantly so (*SI Appendix*, Fig. S5).

Significantly positive postfire ΔQ among heavily burned basins is present in all four seasons (Fig. 3 *B*–*E*). When assessed as standardized anomalies relative to each season’s baseline, these offsets are largest in fall (October to December) and summer (July to September), each averaging nearly +0.60 σ over the first 6 y postfire (Fig. 3 *B* and *E*). Fall and summer are when mean streamflow is lowest in our study basins, allowing a modest runoff boost to register large standardized anomalies. The summer runoff boost is also likely promoted by fire-induced reductions in growing season transpiration and canopy interception.

The +0.38 σ multibasin mean increase in 6-y postfire streamflow translates to a relative increase of 30%. However, relative increases are positively skewed among basins; the multibasin median streamflow increase is 20% (Fig. 3*F*). Notably, 8 of the 29 heavily burned basins do not yet have 6-y of postfire streamflow data. Over the first 2 y postfire, when all burned basins have data, the mean and median increases in water year streamflow are 29% and 19%, respectively, similar to those for 6 y postfire. Despite the large postfire increases in standardized streamflow in fall and summer, the biggest contributor in terms of total water year streamflow is spring; fall is the second biggest contributor (Fig. 3*F*).

**Fig. 4.**
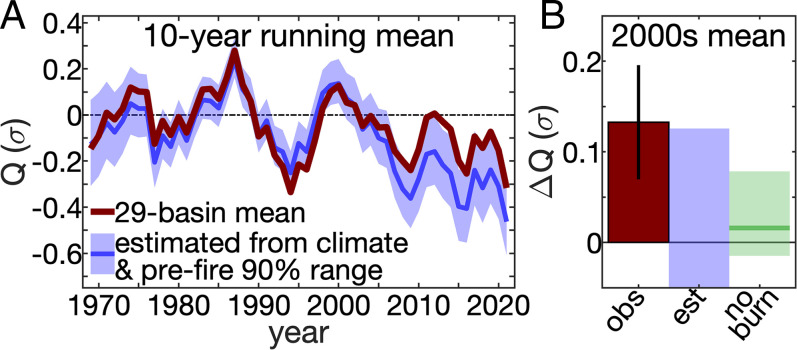
Effect of forest fire on multidecade streamflow in heavily burned basins. (*A*) The 10-y running mean of observed (dark red) and estimated (blue) standardized water year streamflow (Q) anomalies (σ) averaged across 29 heavily burned basins. (*B*) Mean 2000 to 2021 streamflow offset (ΔQ) in heavily burned basins (dark red bar) and 90% confidence interval (vertical black line). Blue areas in *A* and *B* bound the inner 90% of 10,000 repetitions with synthetic time series with prefire variance. Green area in *B* bound the inner 90% of 10,000 repetitions with random unburned basins. Green horizontal line in *B* indicates unburned basin mean.

Rapid recent increases in forest fire activity caused mean 2000 to 2021 water year streamflow among heavily burned basins to be significantly higher (+0.13 σ; *P* < 0.05) than expected from climate alone (Fig. 4 *A* and *B*). Although streamflow has diverged from expectations among heavily burned basins, this effect is not yet detectable at the scale of the entire WUS. Among the 179 forested basins considered here, which contain ∼11% of the total forested area in the WUS, the average 2000 to 2021 water year ΔQ was just +0.02 σ (*SI Appendix*, Fig. S6). This is because the mean 2000s ΔQ was negligible among unburned basins and insignificant among basins where <20% of forest area burned. Our findings that postfire runoff enhancements require roughly >20% of forest area to burn and last an average of 6 y imply that annual burned areas have thus far been too small to substantially affect WUS-wide runoff totals. Just 18% of forested area in our 179 study basins, and 13% of WUS forest area overall, burned during 1984 to 2019.

**Fig. 5.**
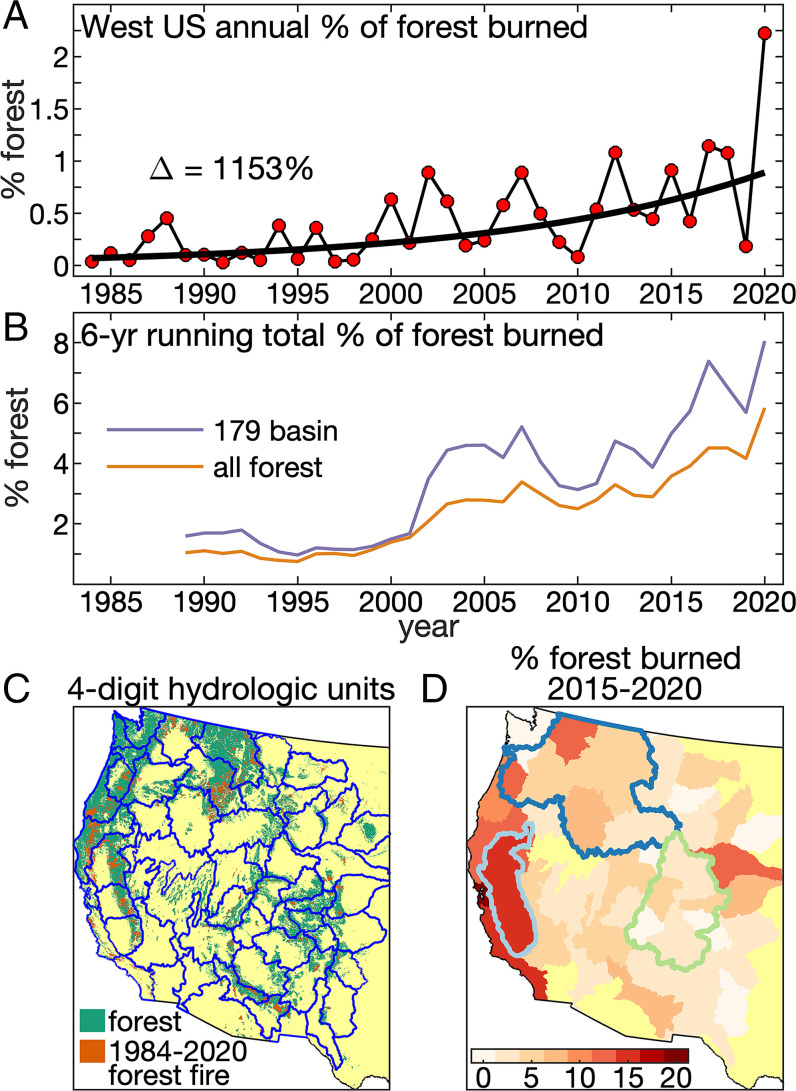
Recent forest fire increases. (*A*) Percentage of WUS forest area burned annually: 1984 to 2020. Trend line calculated by applying the Theil Sen linear trend estimator to the logarithm of percent area burned (Delta is relative trend line change). (*B*) The 6-y running percentage of forest burned in the 179 basins considered in this study (purple) and across the whole WUS (brown). (*C*) Map of boundaries of the WUS four-digit hydrologic units where ≥10% of precipitation falls in forested area overlaid on map of forest (green), 1984 to 2020 burned areas (orange), and nonforest (yellow). (*D*) Map of % forest area burned during 2015 to 2020 within the hydrologic units shown in *B*. Boundaries of three major river basins are overlaid: Sacramento/San Joaquin/Tulare (light blue), Columbia (dark blue), and upper Colorado (green).

Will postfire streamflow boosts continue to be confined to small catchment-level scales? Extending the burned area record through 2020 with a shorter and lower-resolution product (32), we find that the annual fraction of WUS forest area burned in large wildfires grew by ∼1,150% over 1984 to 2020 (Fig. 5*A*). During the final 6 y of this period, 2015 to 2020, 5.8% of WUS forest area burned, ∼6 times the average of all 6-y periods during 1984 to 1999 (Fig. 5*B*). If these trends continue, future annual burned areas will regularly exceed those registered in recent decades. Moreover, increases in burned area do not need to occur across the entire WUS to impact regional water resources. Fig. 5 *C* and *D* shows 2015 to 2020 burned areas at the scale of US Geological Survey (USGS) four-digit hydrologic units, which are much larger than the gauged basins evaluated in our preceding analyses. Recent expansion of forest fire area has been pronounced in watersheds near the US west coast, particularly within the Sacramento, San Joaquin, and Tulare basins that predominantly drain California’s Sierra Nevada range (light blue outline in Fig. 5*D*). In 2015 to 2020, nearly 16% of forest area burned in these basins (Fig. 5*D*), and over 6% of California’s forest area burned in 2020 alone, nearly tripling the previous record from 2018 (*SI Appendix*, Fig. S7). The recent proportion of forest area burned also increased markedly in the Columbia River basin (6% burned in 2015 to 2020) but remained lower in the Upper Colorado River basin (Fig. 5*D* and *SI Appendix*, Fig. S7*A*).

**Fig. 6.**
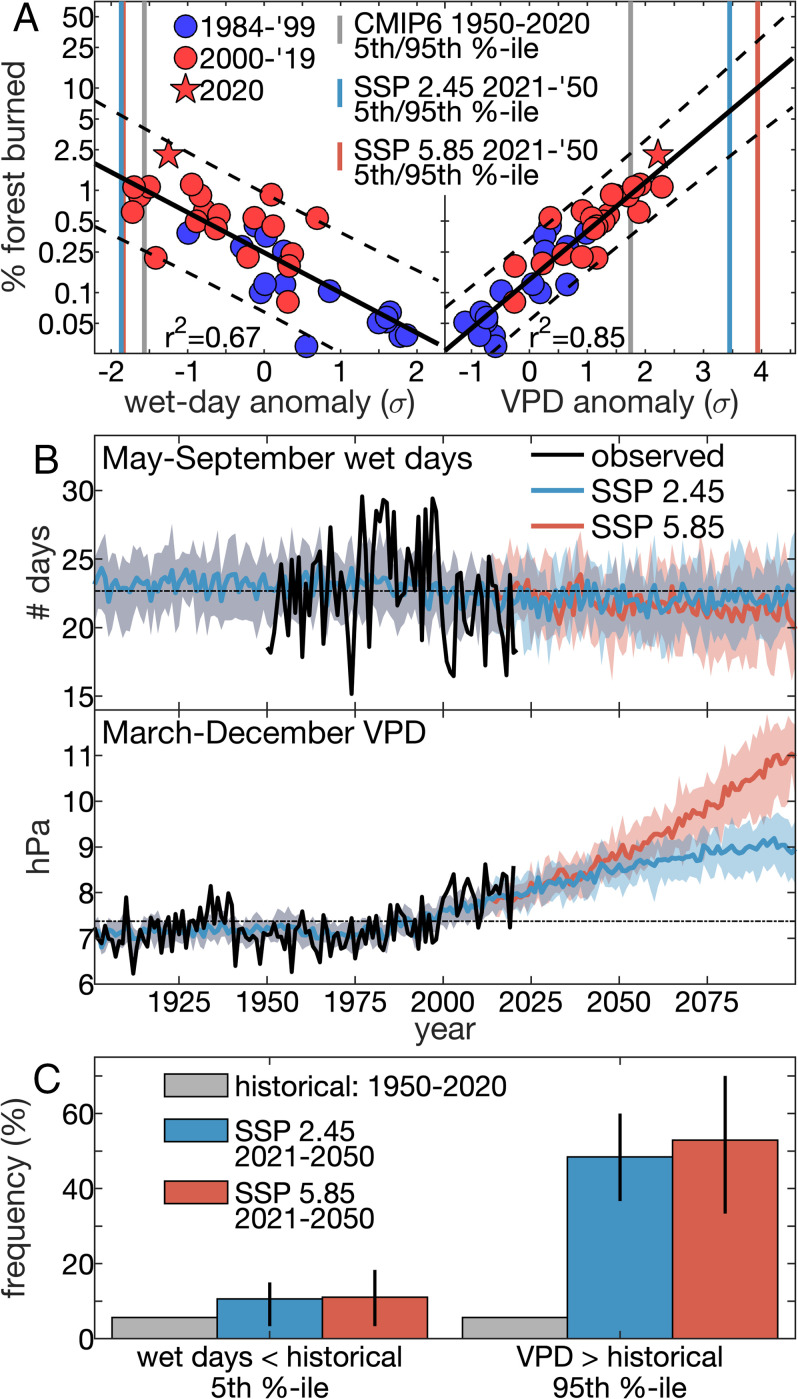
Potential for rapid growth of forest fire area. (*A*) Scatterplot of annual WUS percent forest area burned (log-scale *y* axis) versus standardized anomalies of (*Left*) frequency of May to September wet days and (*Right*) mean March to December VPD overlaid on vertical lines indicating CMIP6 multimodel mean (*Left*) 5th and (*Right*) 95th percentiles calculated over 1950 to 2020 (gray) and 2021 to 2050 (blue and red) for the Shared Socioeconomic Pathway (SSP) 2.45 and SSP 5.85 scenarios, respectively. Black solid and dashed lines indicate observed regression line and 95% prediction interval, respectively (r^2^ values are squared Person’s correlation between each climate variable and the logarithm of annual forest area burned). (*B*) Time series of observed (black) and CMIP6 (blue and red) (*Top*) May to September wet day frequency and (*Bottom*) March to December VPD. (*C*) Frequency of years during 1950 to 2020 (gray) and 2021 to 2050 (blue and red) when models project (*Left*) May to September wet day frequency to be lower than and (*Right*) March to December VPD to be higher than the 5th and 95th percentiles, respectively, of historical (1950 to 2020) variability. In *B* and *C*, CMIP6 values represent multimodel means (bold lines) and interquartiles (shading) for the SSP 2.45 (blue) and SSP 5.85 (red) scenarios.

Previous work established that annual WUS forest fire area responds exponentially to aridity, which we represent in Fig. 6 as a lack of warm season wet days (days with <2.54 mm precipitation) and anomalously high spring–fall atmospheric vapor-pressure deficit (VPD) (14, 16). Throughout 1984 to 2020, the response of annual WUS forest fire area to both of these variables (specifically May to September wet day frequency and March to December VPD) was remarkably strong and stable, and the ongoing rapid increase in forest fire area is consistent with expectations based on observed climate (Fig. 6*A*). For example, the extremely large forest fire area in 2020 was consistent with expectations based on 2020 aridity levels and historical relationships between annual forest fire area and aridity.

Climate models from the sixth phase of the Coupled Model Intercomparison Project (33) simulate future trends in both wet day frequency and VPD that would positively force annual forest fire area. Considering either a high or moderate future emissions scenario (Shared Socioeconomic Pathway 5.85 or 2.45, respectively) (34), climate models project that years exceeding the aridity conditions defined as extreme over 1950 to 2020 will become increasingly common in the coming decades (Fig. 6 *B* and *C*). During 2021 to 2050, models project years with extremely few warm season wet days (1950 to 2020 5th percentile) and extremely high spring–fall VPD (1950 to 2020 95th percentile) to be ∼85% and 750% more frequent than in 1950 to 2020, respectively (Fig. 6*C*).

## Discussion

Following forest fire, streamflow can be enhanced due to reduced vegetation water demand and canopy interception as well as reduced infiltration due to water-repellent soil and/or loss of soil due to erosion (9–12). Averaged across 72 minimally managed, forested basins in the WUS, streamflow was significantly elevated over the first 6 y postfire when compared to that expected from climate alone. A 6-y recovery is short relative to the time needed for forest to return to prefire structure and composition. Possible causes include rapid regrowth of leaf area in the years immediately following fire (23) and loss of fire-induced soil–water repellency on the order of months to several years (35, 36). Importantly, the time required for postfire hydrologic recovery is highly variable (37), and the 6-y period found here is an average that is weighted heavily by basins that burned in the past decade.

Streamflow enhancement was particularly strong following forest fire relative to fire in other vegetation types. Runoff from forested areas may exhibit high sensitivity to altered runoff ratios because forested areas tend to have higher precipitation totals than in shrub and grass vegetation types in the WUS. Forest fire may also have high capacity to drive runoff ratio increases due to loss of canopy interception by large trees and/or high postfire water repellency due to heat and ash.

The postfire streamflow enhancement was detected in all four seasons and strongest in spring and fall. It is perhaps not surprising that the largest streamflow boost occurs in spring, as this is when streamflow rates are highest on average among our basins and also when forest ecosystems may ordinarily begin using soil moisture and therefore when capacity for change due to loss of trees is highest. However, the strong fall signal is surprising because both streamflow and transpiration are on average lower in fall than in spring. Future work to diagnose the high postfire streamflow boost in fall may yield valuable insights as to the dominant mechanisms underlying postfire hydrological change more generally.

Although annual WUS forest fire area grew by over 1,100% over 1984 to 2020, the effects on streamflow are most likely not significant at the large scale of the WUS because the total forest area burned has been too small. Given that postfire streamflow boosts were most consistent among basins that experienced >20% of forest area burned in a single year and that those effects lasted for an average of 6 y, we interpret that a region’s streamflow may be expected to be detectably enhanced if >20% of its forest area burns in a 6-y period. At the scale of the WUS, there has not yet been an occurrence in recent decades of even 10% of WUS forest area burning in 6 y. At regional scales, however, increasing forest fire activity and the exceptional 2020 wildfire season have already brought several large and important basins in California, Oregon, and Washington close to experiencing a 6-y total of 20% of forest area burned.

If the historical relationship between annual forest fire area and aridity remains stable over the next 30 y, then widespread continued increases in forest fire area are very likely, with repeated occurrences of single-year burned areas exceeding that of 2020, which nearly doubled the previous modern record from 2017. Already, as of December 2021, the annual forest area burned in 2021 will be close to that of 2020. Importantly, blind extrapolation of historical fire–climate relationships into a nonanalog future is not wise because fire-critical variables beyond those evaluated here will be important (16, 38); changes in vegetation type and structure due to fire, climate, and humans will modulate future fire–climate relationships (27, 39–41). For example, in addition to growing forest fire extents, WUS forest fire severity has also increased due to warming and drying (17), and capacity for postfire forest recovery is increasingly challenged in many areas due to aridification (8, 42, 43). Long-term loss of forest cover would likely contribute additional runoff effects beyond those expected from increases in burned area alone but may also weaken the potent ability of aridity to drive rapid increases in forest fire. Nonetheless, the exponential relationship between WUS forest fire area and aridity was remarkably consistent over the past 4 decades, and 2020 provided the latest example of how annual forest fire areas can massively increase to remain consistent with historical fire–climate relationships.

More research is needed to project future changes in wildfire and streamflow effects confidently, but our results suggest that increasing forest fire activity is unhinging WUS streamflow from its historically predictable response to climate variability. Importantly, the postfire streamflow enhancements we observed are relative to expectations based on climate and should not be misinterpreted as absolute increases in streamflow. In fact, streamflow declined over 1970 to 2021 in the majority of our study basins. Warming has likely contributed to these trends by enhancing evaporative demand (44, 45). When only viewed through the lens of water limitation, the prospect for increased streamflow may be seen as a positive outcome, but increases in postfire runoff, particularly after severe fire, are often accompanied by large sediment loads; reduced water quality; and enhanced flood, debris flow, and landslide hazards (12, 13, 46, 47). Increased runoff throughout the year may also challenge water management efforts to optimize reservoir storage while maintaining capacity to accommodate large runoff pulses in winter and spring (48), particularly as warming promotes ever-increasing rain-to-snow ratios and earlier snowmelt. Wildfire is currently not factored into assessments of climate change effects on WUS streamflow, but our findings suggest that fire will soon come to play an important role in the changing hydrology of this water-limited region.

## Materials and Methods

### Data.

All data are publicly available. Sources are listed in *SI Appendix*, Table S1, and data preparation methods are described in *SI Appendix*, Supplementary Text S2. A list of Coupled Model Intercomparison Project Phase 6 (CMIP6) models used for projections of wet day frequency and VPD is provided in *SI Appendix*, Table S2.

### Study Basins.

We used daily streamflow data from 179 USGS gauges located in the WUS with near-continuous coverage over at least 1975 to 2020 and on streams that drain geographically distinct basins that are ≥10 km^2^ in area, >25% forested, and classified as minimally disturbed by people. Seventy-two basins were classified as burned because during 1984 to 2019, >5% of the basin area, including at least some forest area, burned in at least 1 y. In each burned basin, the primary fire year was when the greatest proportion of the basin’s area burned. The other 107 basins were classified as unburned. Forest was the dominant land cover type among the study basins, averaging 76% coverage per basin. Nonforested areas were generally classified as shrub or grass, averaging 15% and 5%, respectively. Distributions of land cover classifications were similar between the burned and unburned basins. Additional details about gauge and basin selection and classification of burned basins are in *SI Appendix*, Supplementary Text S1. *SI Appendix*, Supplementary Text S2 describes the land cover classification methods. *SI Appendix*, Table S3 lists each basin’s gauge identification number, gauge location, and fire year.

### Modeled Streamflow and Effects of Wildfire.

For each burned basin, we developed a model that uses seasonal climate to estimate water year runoff ratio (fraction of the basin’s total water year precipitation that flows past the gauge during the water year). For burned basins, we estimated water year runoff ratios for 1960 to 2021 based on prefire relationships with seasonal climate (see Fig. 1*B* for example). We then estimated water year streamflow by multiplying estimated runoff ratio by each basin’s water year precipitation rate. Potential predictors of runoff ratio were seasonal precipitation total, logarithm of precipitation total, square of precipitation total, tmax, and ETo. Seasons considered were October to December (OND), January to March (JFM), April to June (AMJ), and July to September (JAS) of the corresponding water year (climate conditions in preceding water years were not considered). In addition to considering each climate variable in each season, we considered climate conditions over all six combinations of consecutive seasons in the water year (ONDJFM, ONDJFMAMJ, ONDJFMAMJJAS, JFMAMJ, JFMAMJJAS, and AMJJAS). Thus, each of five climate variables had 10 opportunities for consideration in each model (50 potential predictors). To reduce overfitting, we constructed the models in a stepwise fashion. The first predictor was the variable with the strongest (maximum absolute) Pearson’s correlation with runoff ratio. Runoff ratio was then estimated using linear regression, and model skill was assessed with the Akaike information criterion with a bias correction for small sample size (AICc), which penalizes for additional predictor variables. A time series of residual runoff ratios was then calculated, the predictor variable was identified that most strongly correlated with the residuals, and that predictor variable was combined with the first predictor in a multiple regression to estimate runoff ratio. If the new multiple regression reduced the original AICc value by more than 2 (49), the new predictor variable was retained and the process continued.

Each burned basin’s runoff ratio model was trained only on data prior to the wildfire year and then applied to estimate water year streamflow for the full 1960 to 2021 period. We calculated Q_est_ by standardizing the time series of estimated streamflow values relative to 1970 to 2000, the period of maximal prefire data coverage among gauges, to promote comparability among basins. Each basin’s corresponding time series of Q_obs_ was rescaled to match the mean and variance of Q_est_ during prefire years. Streamflow offsets from expectations (ΔQ) are Q_obs_ − Q_est_.

### Significance Testing.

We evaluated significance of ΔQ values by comparing to 1) time series of ΔQ from unburned basins and 2) synthetic time series of ΔQ based on prefire variability. The hypothesis was tested that postfire ΔQ is higher than 95% of values when analyses are repeated with 10,000 alternative records in which ΔQ is unaffected by wildfire. We interpreted postfire ΔQ as significantly positive if this one-tailed *P* < 0.05 test was passed for both methods, which are described below.

#### Method 1: Comparison to unburned basins.

In each of 10,000 iterations, each burned basin’s time series of ΔQ was replaced by an alternative time series of ΔQ from a randomly drawn unburned basin. In each case, years of missing streamflow data in the burned basin were assigned as missing for the unburned basin, and the unburned basin’s runoff ratio model was parameterized based only on years prior to the burned basin’s fire year.

#### Method 2: Comparison to prefire variability.

In each of 10,000 iterations, each burned basin’s time series of ΔQ was replaced with a synthetically generated time series of ΔQ that is characterized by the observed prefire ΔQ variability. Each basin’s runoff ratio model was parameterized on prefire data only, so postfire Q_est_ is out of sample, which should automatically enhance ΔQ variability postfire. Therefore, for each basin, we generated a second time series of ΔQ based only on out-of-sample estimates (ΔQ_oos_). For basins with no significant lag-1 autocorrelation in the originally calculated time series of prefire ΔQ, we assumed years to be independent and calculated the out-of-sample estimates of prefire streamflow (Q_est_oos_) by withholding a single year of data at a time. When lag-1 autocorrelation was significant, we withheld multiyear windows of prefire data, following Wilks (50) to calculate window length for independent sampling. Basins with significant autocorrelation in prefire ΔQ were those where lag-1 autocorrelation was significant at the *P* < 0.05 level after adjusting to account for the false discovery rate expected when assessing many correlation values (51).

Within each of the 10,000 sets of synthetic time series of ΔQ_oos_ for the 72 burned basins, we retained the spatiotemporal correlation structure evident among observed records of ΔQ_oos_. We retained spatial correlation through a principal components approach. We performed a principal components analysis based on the 179 × 179 correlation matrix calculated from records of ΔQ_oos_ from all burned and unburned basins during 1970 to 2000. In calculating the correlation matrix, all available prefire years during 1970 to 2000 were considered for each pair of basins. We then calculated 179 principal component time series (PCs) by applying the resultant empirical orthogonal function (EOF) loading coefficients to the observed records of prefire ΔQ_oos_ after standardizing relative to all available prefire years during 1970 to 2000. Because ΔQ_oos_ values were not available for all basins in all years, each year’s PC values were scaled by multiplying by 179 divided by the number of basins with prefire ΔQ_oos_ data. Because the correlation matrix was based on just 1970 to 2000, nearly all temporal variability in the standardized records of ΔQ_oos_ was concentrated in the first 30 PC time series. For each of these 30 PCs, we saved the variance and low-order autoregressive coefficients, both calculated over 1970 to 2000. We also saved, for each basin, the additional variance in the observed record of ΔQ_oos_ not accounted for by the first 30 PCs as well as any low-order autoregressive coefficients associated with this residual variability in ΔQ_oos_.

Each of the 10,000 sets of synthetic records of ΔQ_oos_ was based on 30 random time series of PCs with the observed PCs’ variances and autoregressive structures. For each of the 72 burned basins in each of the 10,000 simulations, we calculated a synthetic record of random ΔQ_oos_ by 1) using the 30 synthetic PCs and the observed EOF coefficients to calculate a synthetic record of the PC-derived portion of that basin’s standardized record of ΔQ_oos_, 2) calculating a separate random time series with the observed variance and autoregressive structure of the observed portion of the basin’s standardized record of ΔQ_oos_ that was not associated with the first 30 PCs, 3) adding these two PC and non-PC time series together, and 4) multiplying by the observed 1970 to 2000 ΔQ_oos_ variance.

### Seasonal Analysis.

In addition to water year analyses, a record of ΔQ was developed for each basin and season (OND, JFM, AMJ, and JAS). Whereas water year streamflow was represented as the product of runoff ratio and precipitation over the full water year, the part of the year when precipitation influences seasonal streamflow varies by season and among basins. For each basin and season, we first found the range of 3 to 12 consecutive months (during the 12 mo that end in the final month of each season) when precipitation total correlated most strongly with seasonally averaged streamflow during prefire years. The precipitation total during this range of months was then treated as the primary predictor of seasonal runoff, to be multiplied by a modeled time series of runoff ratio for that basin and season. Modeled records of seasonal runoff ratio were developed following the methods used to model water year runoff ratio except that seasonal climate predictors were selected over a 12-mo window ending in the final month of each season rather than the final month of the water year. For each basin, the four seasonal records of ΔQ were rescaled so they averaged to equal the previously calculated record of water year ΔQ.

## Supplementary Material

Supplementary File

## Data Availability

Previously published data were used for this work (all data are publicly available, and sources are listed in *SI Appendix*, Table S1).
