# Supplementary material for: Growing impact of wildfire on western US water supply
Source: Proc Natl Acad Sci U S A. 2022 Feb 22;119(10):e2114069119. doi: 10.1073/pnas.2114069119 (PMC8915835; doi:10.1073/pnas.2114069119)
Supplement: Supplementary File [file pnas.2114069119.sapp.pdf]

Supplementary Information for

Growing impact of wildfire on western United States streamflow

A. Park Williams[a,b,*], Ben Livneh[c,d], Karen A. McKinnon[e,f], Winslow D. Hansen[g], Justin S. Mankin[b,h], Benjamin I. Cook[b,i], Jason E. Smerdon[b], Arianna M. Varuolo-Clarke[b,j], Nels R. Bjarke[d], Caroline S. Juang[b,j], Dennis P. Lettenmaier[a]

[a]Department of Geography; University of California, Los Angeles, Los Angeles, CA
[b]Lamont-Doherty Earth Observatory of Columbia University, Palisades, NY
[c]Cooperative Institute for Research in Environmental Sciences; University of Colorado Boulder, Boulder, CO
[d]Civil, Environmental and Architectural Engineering; University of Colorado Boulder; Boulder, CO
[e]Department of Statistics and Institute of the Environment and Sustainability; University of California, Los Angeles; Los Angeles, CA
[f] Institute of the Environment and Sustainability; University of California, Los Angeles; Los Angeles, CA
[g]Cary Institute of Ecosystem Studies; Millbrook, NY
[h]Department of Geography; Dartmouth College; Hanover, NH
[i]NASA Goddard Institute for Space Studies; New York, NY
[j]Department of Earth and Environmental Sciences, Columbia University; New York, NY

[1]corresponding author: williams@geog.ucla.edu

This PDF file includes:

Supplementary Text S1 to S2
Tables S1 to S3
Figures S1 to S7
SI References

Supplementary Text S1: Streamflow gauge selection

We downloaded daily streamflow data for water years 1960–2021 for the 293 United States Geological Survey (USGS) gauges that met the following criteria: (i) west of 102°W, (ii) downstream of a basin ≥10 km^2 in area, (iii) has a USGS GAGES-II hydro-disturbance classification of <10 to assure low anthropogenic modification of the basin, (iv) ≤1 major upstream dam (>5000 acre-feet) with percent reservoir storage <10% of 1971–2000 mean annual streamflow, (v) active as of 2017, and (vi) streamflow data for ≥80% of days during each half of water years 1975–2020. Prior to this assessment of daily data coverage we performed simple gap filling where missing streamflow values for ≤10 consecutive days were replaced with linearly interpolated values based on the days prior to and following the gap.

For each of the 293 gauges we calculated monthly mean streamflow for 1960–2021. If a monthly value was missing but immediately preceded or followed by a complete month of data, we performed gap-filling based on other gauges (thus gap filling was not applied across >2 consecutive months). For a gauge with ≥1 missing value for month *j* (e.g., March), alternate gauges were considered for gap-filling if they had ≥20 overlapping values with the target gauge for month *j* and the Pearson's correlation was ≥0.85. When these criteria were met by ≥1 gauge, gap-filling was performed using quantile mapping prioritizing alternate gauges in order of descending correlation.

Among the gauges considered up to this point, some did not represent entirely unique basins, were not forested, or had very low flows. In cases where basins overlapped, only the gauge associated with the most heavily forested (based on % forest coverage) basin was retained, leaving 266 gauges associated with unique basins. We further eliminated 68 gauges: 29 gauges associated with basins with ≤25% forest coverage (due to our specific focus on forest fire), 24 gauges with zero flow for at least one water year or a long-term mean water-year flow of ≤10 cubic feet per second (due to potential for very small changes in runoff to register large relative anomalies), and 15 gauges with poor correlation ($r^2 < 0.5$) between water-year runoff and water-year precipitation (suggesting that non-climate factors dominate streamflow variability), leaving 198 gauges. In the assessment of correlation between water-year runoff and precipitation, this correlation was calculated for only pre-fire years for gauges associated with burned basins.

We classified a basin as "burned" if during 1984–2019, >5% of the basin area burned in at least one year, including at least some forest area. In each burned basin, the "fire year" was identified as the year when the greatest proportion of the basin's area burned. Burned basins were discarded if there were <15 complete water years of streamflow data prior to the fire year (for characterization of pre-fire response of streamflow to climate) or <2 complete water years of streamflow data after the fire year (for characterization of streamflow response to fire). We classified a basin as "unburned" if no year in 1984–2019 exhibited a co-occurrence of >5% of total area burned and >0% of forest area burned. However, some basins classified as "unburned" did experience a cumulative forest area burned of >5% during the study period. This resulted in 72 burned basins and 123 unburned basins, with 3 basins discarded for not meeting criteria for either classification. There were 195 gauges remaining after this step.

To promote comparability between burned and unburned basins, we discarded 16 unburned basins that experienced 1981–2010 climate normals for either mean annual precipitation or mean annual temperature (average of tmax and tmin) that were beyond the minimum or maximum values experienced among burned basins (burned basin ranges of mean annual precipitation and

temperature: 473–2955 mm, 4.5–24.4°C). This left us with a total of 179 gauges and associated basins: 72 burned and 107 unburned (Fig. 1a).

Finally, the high-resolution, satellite-based maps that we used to map burned areas in our study basins only extend through 2019, but our study's finding that streamflow tends to be enhanced in the years immediately following wildfire indicates that our study's results may be affected by the extensive wildfire season in 2020. For each of the 179 study basins, we produced an alternate record of annual area burned for 2001–2020 using the MODIS burned area product described in Supplementary Text S2. This product has a coarse spatial resolution (500 m) relative to that of the 30 m MTBS dataset, which causes the MODIS binary determination of burned grid cells to often over-represent true within-cell burned area. We bias-corrected all MODIS records of basin-specific annual burned areas by, for each basin, multiplying the MODIS annual burned areas by the mean ratio of MTBS-to-MODIS annual burned areas during their 2001–2019 period of overlap. We then determined using the bias-corrected MODIS burned areas that 2020 would have qualified as the fire year for 3 basins classified as unburned and 5 basins classified as burned. Rather than treat 2020 as the fire year for these basins in our analyses, which would allow for only a single year of post-fire observations and introduce high uncertainty in area burned due to the low resolution of the MODIS data, we simply replaced all streamflow data in 2021, the first year when we would expect post-fire streamflow effects, with NaN values for these basins.

Supplementary Text S2: Dataset details

Daily streamflow data are from the USGS Gages II database. Each gauge has an 8-digit hydrologic unit code corresponding to its upstream watershed. To access daily data for a given gauge, we used the following URL formula:

```
https://nwis.waterdata.usgs.gov/nwis/?cb_00060=on&format=rdb&site_no=GAGE_ID
&referred_module=sw&period=&begin_date=YYYY-MM-DD&end_date=YYYY-MM-DD
```

where `GAGE_ID` is replaced with the 8-digit hydrologic unit code, the first instance of `YYYY-MM-DD` is replaced with the first date of desired gauge data and the second instance of `YYYY-MM-DD` is replaced with the final date of desired gauge data.

Gauge locations, coordinates of basins boundaries, and gauge and basin attributes are from the USGS GAGES II Geospatial Attributes of Gages for Evaluating Streamflow "Basin characteristics and update report." Basins were mapped onto a 1-km equal-area grid for the WUS; for each basin the fractional overlap was calculated for each grid cell.

Forest coverage was determined from the USGS National Land Cover Database (NLCD). The NLCD provides 30-m maps of landcover classifications across the coterminous US for 1992, 2001, 2004, 2006, 2008, 2011, 2013, and 2016, which we aggregated to 1-km and 12-km maps of fractional forest cover. We then produced a master map of forest coverage at both resolutions: for each grid cell we defined forest coverage as the maximum value among the 8 NLCD years to best represent pre-fire conditions. For each basin we calculated the area-weighted mean fractional forest coverage from the 1-km grid.

Wildfire data are from the US Forest Service's Monitoring Trends in Burn Severity (MTBS) database (1). This satellite-derived, 30-m gridded dataset classifies burn severity for large wildfires ≥404 ha (4.04 km^2) in area for 1984–2019. According to data from Short et al. (2),

wildfires ≥404 ha represent less than 2% of fires in WUS forested areas, but comprise approximately 97% of the total area burned. We considered wildfire area to be represented by any of the 3 MTBS severity classes (mild, moderate, and severe) and aggregated the 30-m imagery to 1-km maps of annual area burned for 1984–2019. For each basin we calculated annual total area burned and total forest area burned. For calculation of annual forest area burned across the entire WUS, we extended the record through 2020 using the version 6 MODIS burned-area product, which covers 2001–2020 (3). The record of annual MODIS forest area burned was linearly adjusted to best match the MTBS record during the 2001–2019 period of overlap.

For analyses of fire severity and stand-replacing fire, we used 30-m Landsat-derived maps of burn severity for MTBS fires during 1985–2017 from Parks and Abatzoglou (4). This dataset includes corresponding maps of satellite measurements of the normalized difference vegetation index (NDVI). Following Parks and Abatzoglou, grid cells with NDVI<0.35 were masked out as non-forest and fire severity values ≥2.25 were assessed as stand-replacing fire. We aggregated the 30-m data to produce 1-km maps of mean fire severity and area of stand-replacing fire.

We followed the methods of Williams et al. (5) to produce monthly grids of total precipitation and mean daily maximum and minimum temperature (tmax and tmin, respectively), saturation vapor pressure (e_s), actual vapor pressure (e_a), wind speed, solar radiation, and reference evapotranspiration (ETo) from January 1901 through September 2021 at a 12-km equal-area resolution across the WUS. The employed climate datasets were the National Oceanic and Atmospheric Administration (NOAA) Climgrid product (6), TopoWx (7), PRISM (8), NLDAS2 (9), and the Princeton Global Forcing dataset (10). Monthly Penman-Monteith ETo (11) was calculated from precipitation, tmax, tmin, e_s, e_a, wind speed, and solar radiation using the United Nations Food and Agriculture Organization formulation assuming well-watered short-grass conditions (12). As atmospheric carbon dioxide concentration ([CO_2]) increases, the amount of water required by plants per unit photosynthesis should reduce (13), all else being equal. We attempted to account for this by reducing surface resistance in the ETo calculation as a function of [CO_2] following Yang et al. (14).

Monthly precipitation frequency (days with non-zero precipitation) and wet-day frequency (days with ≥2.54 mm precipitation) were calculated from 1/24° daily precipitation grids from Gridmet from January 1979 through September 2021 and aggregated to 12-km resolution (15). To extend the record of precipitation frequency back to 1950 we developed an alternate 12-km product from daily station data from the Global Historical Climatology Network (GHCN) for 1950–2020 following methods from Borkotoky et al. (16).

The 12-km map of fractional forest coverage was used to calculate monthly forest-area weighted means of March–December VPD and May–September wet-day frequency for the WUS. The Gridmet-based record of wet-day frequency was extended back to 1950 by calibrating the GHCN-based record to have the mean and variance of the Gridmet record during their 1979–2020 period of overlap.

For streamflow modeling we used monthly records of precipitation total, tmax, tmin, and ETo for each basin, calculated by linearly interpolating the 12-km climate grids to 1-km resolution and calculating area-weighted spatial means based on overlapping 1-km grid cells for each basin. For precipitation, an adjustment was made prior to calculation of each basin's monthly total to account for fine-scale topography: 1/120° maps of 1981–2010 monthly mean

precipitation from PRISM were re-gridded to 1-km and 12-km monthly precipitation totals were re-scaled to match the high-resolution PRISM climatological means.

For climate-model simulations of historical and future wet-day frequency and VPD we used daily outputs of precipitation totals and monthly outputs of near-surface tmax, tmin, and relative humidity for the CMIP6 historical (1850–2014) scenario and two future (2015–2100) scenarios, the SSP2.45 and SSP5.85. For wet-day frequency, the necessary daily data for the three scenarios were available for 32 models. For VPD, the necessary monthly data for the three scenarios were available for 34 models. See Table S2 for a list of models used (for some models, Tmax and Tmin were not available so we used mean average temperature for these models). We considered a single simulation output (run) per model because daily CMIP6 outputs are data intensive. For each model run and scenario, we calculated May–September wet-day frequency and March–December VPD at the model's native resolution, re-gridded to 12-km resolution using nearest-neighbor interpolation, and calculated forest-area weighted means for the WUS. For each model, variable, and future emissions scenario we then produced a full time series for 1850–2100 by appending the future-scenario time series (2015–2100) to the historical time series (1850–2014). Finally, we standardized all time series relative to 1950–2020 and rescaled to the observed 1950–2020 mean and variance for comparison to observations.

For each basin, mean elevation and slope came from the 1/120° NOAA Global Land One-kilometer Base Elevation dataset (17).

Supplementary Tables and Figures

Supplementary Table S1. Sources of dataset used

Dataset	URL and date accessed
USGS daily stream gauge data	https://nwis.waterdata.usgs.gov/nwis<br>Accessed December 23, 2021
GAGES-II: Geospatial Attributes of Gages for Evaluating Streamflow	https://doi.org/10.3133/70046617<br>Accessed April 22, 2021
US Forest Service Monitoring Trends in Burn Severity maps of burned area	https://www.mtbs.gov/direct-download<br>Accessed September 29, 2021
MODIS version 6 Burned Area Product	Download information available at https://modis-fire.umd.edu/files/MODIS_C6_Fire_User_Guide_C.pdf<br>Accessed December 22, 2021
Parks & Abatzoglou (2020) burn severity and NDVI	https://datadryad.org/stash/dataset/doi:10.5061/dryad.tmpg4f4x1<br>Accessed January 30, 2021
NOAA Climgrid climate data	https://doi.org/10.7289/V5SX6B56<br>Accessed December 8, 2021
TopoWx v1.3.0 temperature data	http://www.scrimhub.org/resources/topowx<br>Accessed July 7, 2017
PRISM version M3 climate data	www.prism.oregonstate.edu<br>Accessed December 2, 2021
National Land Data Assimilation System version 2 climate forcing data	https://hydro1.gesdisc.eosdis.nasa.gov/data/NLDAS<br>Accessed December 8, 2021
Princeton Global Forcing version 2 climate dataset	http://hydrology.princeton.edu/data/pgf/v2/0.5deg<br>Accessed May 12, 2016
Princeton Global Forcing version 3 climate dataset	http://hydrology.princeton.edu/data/pgf/v3/0.25deg<br>Accessed May 9, 2018
Gridmet daily climate data	www.climatologylab.org/gridmet.html<br>Accessed December 23, 2021
Global Historical Climatology Network daily precipitation data	https://www.ncdc.noaa.gov/ghcnd-data-access<br>Accessed October 9, 2020
CMIP6 climate model simulation outputs	https://esgf-node.llnl.gov/search/cmip6/<br>Accessed June 8, 2021
Landcover classifications from the USGS National Land Cover Database	https://www.mrlc.gov/data<br>Accessed October 4, 2020
NOAA Global Land One-kilometer Base Elevation dataset	https://www.ngdc.noaa.gov/mgg/topo/gltiles.html<br>Accessed September 2, 2020

Supplementary Table S2. CMIP6 models and runs used. For wet-days, daily precipitation (pr) totals were used. For VPD, monthly means of relative humidity (hurs), daily maximum temperature (tasmax), and daily minimum temperature (tasmin) were used unless the fourth column indicates "tas", in which case daily average temperature was used instead of tasmax and tasmin.

Model	Wet-days run	VPD run	tasmax/tasmin or tas
ACCESS-CM2	r1i1p1f1	r1i1p1f1	tasmax/tasmin
ACCESS-ESM1-5	r1i1p1f1	r1i1p1f1	tasmax/tasmin
BCC-CSM2-MR	r1i1p1f1	N/A	N/A
CAMS-CSM1-0	r2i1p1f1	N/A	N/A
CESM2	r4i1p1f1	r10i1p1f1	tas
CESM2-WACCM	r1i1p1f1	r1i1p1f1	tas
CMCC-CM2-SR5	r1i1p1f1	r1i1p1f1	tas
CNRM-CM6-1	r1i1p1f2	r1i1p1f2	tasmax/tasmin
CNRM-CM6-1-HR	N/A	r1i1p1f2	tasmax/tasmin
CNRM-ESM2-1	r1i1p1f2	r1i1p1f2	tasmax/tasmin
CanESM5	r1i1p1f1	r1i1p1f1	tasmax/tasmin
CanESM5-CanOE	N/A	r1i1p2f1	tasmax/tasmin
EC-Earth3	r1i1p1f1	r1i1p1f1	tasmax/tasmin
EC-Earth3-Veg	r1i1p1f1	r1i1p1f1	tasmax/tasmin
EC-Earth3-Veg-LR	r1i1p1f1	r1i1p1f1	tasmax/tasmin
FGOALS-f3-L	N/A	r1i1p1f1	tas
FGOALS-g3	r1i1p1f1	r1i1p1f1	tasmax/tasmin
FIO-ESM-2-0	N/A	r1i1p1f1	tasmax/tasmin
GFDL-CM4	r1i1p1f1	r1i1p1f1	tasmax/tasmin
GFDL-ESM4	r1i1p1f1	r1i1p1f1	tasmax/tasmin
GISS-E2-1-G	N/A	r1i1p3f1	tasmax/tasmin
HadGEM3-GC31-LL	r1i1p1f3	r1i1p1f3	tasmax/tasmin
IITM-ESM	r1i1p1f1	r1i1p1f1	tas
INM-CM4-8	r1i1p1f1	r1i1p1f1	tasmax/tasmin
INM-CM5-0	r1i1p1f1	r1i1p1f1	tasmax/tasmin
IPSL-CM6A-LR	r1i1p1f1	r1i1p1f1	tasmax/tasmin
KACE-1-0-G	r1i1p1f1	r1i1p1f1	tasmax/tasmin
KIOST-ESM	r1i1p1f1	r1i1p1f1	tas
MCM-UA-1-0	N/A	r1i1p1f2	tas
MIROC-ES2L	r1i1p1f2	r1i1p1f2	tasmax/tasmin
MIROC6	r1i1p1f1	r1i1p1f1	tasmax/tasmin
MPI-ESM1-2-HR	r1i1p1f1	r1i1p1f1	tasmax/tasmin
MPI-ESM1-2-LR	r1i1p1f1	r1i1p1f1	tasmax/tasmin
MRI-ESM2-0	r1i1p1f1	r1i1p1f1	tasmax/tasmin
NESM3	r1i1p1f1	N/A	N/A
NorESM2-LM	r1i1p1f1	N/A	N/A
NorESM2-MM	r1i1p1f1	r1i1p1f1	tas
UKESM1-0-LL	r1i1p1f2	r1i1p1f2	tasmax/tasmin

Supplementary Table S3. USGS Gauges used

Gauge ID	Latitude	Longitude	Fire Year	Gauge ID	Latitude	Longitude	Fire Year
05014500	48.7991	-113.6568	unburned	11468000	39.1721	-123.6695	unburned
06019500	45.1924	-112.1425	unburned	11468500	39.4282	-123.7378	unburned
06043500	45.4974	-111.2705	unburned	11469000	40.3132	-124.2837	unburned
06093200	48.37	-112.8028	2015	11473900	39.7063	-123.3253	2012
06192500	45.5972	-110.566	1988	11475560	39.7296	-123.6439	unburned
06224000	43.1769	-109.2029	2012	11476600	40.3513	-124.0039	unburned
06280300	44.2083	-109.5549	2013	11478500	40.4804	-123.8909	2015
06289000	45.0069	-107.6151	unburned	11481200	41.011	-124.0817	unburned
06298000	44.8494	-107.3045	unburned	11482500	41.2993	-124.0512	unburned
06311000	44.0277	-107.0809	unburned	11521500	41.8351	-123.3831	2018
06422500	44.1439	-103.4549	unburned	11522500	41.3776	-123.4776	1987
06622700	41.3702	-106.5206	unburned	11523200	41.1113	-122.7056	unburned
06623800	41.0236	-106.8248	unburned	11528700	40.6499	-123.4942	2015
07207500	36.5736	-104.9467	2002	11532500	41.7915	-124.0762	2002
07215500	35.9409	-105.2503	unburned	12013500	46.6509	-123.6527	unburned
08267500	36.5417	-105.5564	unburned	12020000	46.6173	-123.2776	unburned
08271000	36.5084	-105.5308	unburned	12025000	46.6201	-122.9451	unburned
08279000	36.2109	-105.9136	unburned	12025700	46.7726	-122.594	unburned
08291000	35.9647	-105.9045	2013	12045500	48.0548	-123.5832	unburned
08324000	35.6617	-106.7434	2011	12048000	48.0143	-123.1327	unburned
08378500	35.7084	-105.6825	2013	12054000	47.684	-123.0116	unburned
08380500	35.652	-105.3189	unburned	12082500	46.7526	-122.0837	unburned
09026500	39.91	-105.8783	unburned	12092000	46.9037	-122.0351	unburned
09036000	39.8339	-106.0564	unburned	12115000	47.3701	-121.6251	unburned
09059500	39.8	-106.5839	unburned	12134500	47.8373	-121.6668	unburned
09065500	39.6258	-106.2781	unburned	12143400	47.4151	-121.5873	unburned
09066200	39.6483	-106.3231	unburned	12167000	48.2615	-122.0476	unburned
09081600	39.2322	-107.2273	unburned	12175500	48.6726	-121.0729	unburned
09124500	38.2989	-107.2301	unburned	12205000	48.906	-121.8443	unburned
09210500	42.0961	-110.4166	2000	12302055	48.3555	-115.3149	unburned
09223000	42.1105	-110.7096	unburned	12324590	46.5197	-112.7934	unburned
09255000	40.9825	-107.3828	unburned	12330000	46.4721	-113.234	unburned
09266500	40.5775	-109.6221	unburned	12332000	46.1845	-113.5025	2017
09277500	40.3002	-110.6024	unburned	12344000	45.9721	-114.1415	2000
09289500	40.6066	-110.5271	unburned	12358500	48.4952	-114.0101	2003
09292500	40.5119	-110.3415	unburned	12370000	48.0244	-113.9798	unburned
09312600	39.8758	-111.0374	unburned	12389500	47.5919	-115.2296	2007
09342500	37.2661	-107.0109	2013	12390700	47.586	-115.3552	unburned
09404450	37.3394	-112.6044	2012	12409000	48.5943	-118.0625	unburned
09430600	33.1667	-108.6498	2012	12411000	47.7061	-115.9792	unburned
09492400	33.8223	-109.8145	unburned	12414500	47.2746	-116.189	unburned
09496500	33.9859	-110.2809	2002	12414900	47.1763	-116.4927	unburned
09497800	33.8431	-110.5576	2002	12447390	48.8229	-120.1459	2003
09497980	33.8278	-110.8562	2016	12451000	48.3296	-120.6918	unburned
09499000	33.98	-111.3035	2004	12452800	47.8185	-120.4231	2015
09504500	34.7645	-111.891	2014	12459000	47.5832	-120.6195	unburned
09505200	34.6747	-111.6721	unburned	12488500	46.9776	-121.1687	2017
09505350	34.7286	-111.7757	2014	13011500	43.8503	-110.5178	1988
09505800	34.5386	-111.694	2016	13011900	43.8381	-110.4411	2012

09507980	34.2764	-111.6388	2004	13018300	43.4522	-110.7041	unburned
09508300	34.1609	-111.6929	2004	13023000	43.1428	-110.9767	unburned
09510200	33.6942	-111.5418	2005	13047500	44.0686	-111.2414	1988
10032000	42.2933	-110.8724	unburned	13120000	43.9336	-114.1125	unburned
10109000	41.7433	-111.7827	unburned	13185000	43.6594	-115.7272	1994
10150500	40.0497	-111.548	2018	13186000	43.4958	-115.3081	2012
10173450	37.6228	-112.5169	2017	13200000	43.6481	-115.9897	unburned
10194200	38.5791	-112.2902	2010	13235000	44.0853	-115.6222	1989
10205030	38.9119	-111.5305	unburned	13240000	44.9136	-115.9972	1994
10215900	39.2591	-111.5799	unburned	13258500	44.5794	-116.6433	2018
10242000	37.6722	-113.0347	unburned	13313000	44.9617	-115.5	2007
10263500	34.4208	-117.8395	2002	13331500	45.6199	-117.7266	unburned
10296500	38.5132	-119.4499	2002	13336500	46.0867	-115.5139	2012
10308200	38.7146	-119.7649	2015	13337000	46.1508	-115.5872	2012
10310000	38.7696	-119.8338	unburned	13338500	46.0864	-115.9767	unburned
10316500	40.6908	-115.477	2018	13340600	46.8405	-115.6207	unburned
10336660	39.1074	-120.1621	unburned	13345000	46.9152	-116.951	unburned
10336676	39.1321	-120.1577	unburned	14013000	46.0079	-118.1186	unburned
10336780	38.9199	-119.9724	unburned	14020000	45.7196	-118.3233	unburned
10343500	39.4316	-120.238	unburned	14097100	44.8565	-121.1498	1996
10396000	42.7908	-118.8675	1999	14137000	45.3996	-122.1373	unburned
11055800	34.1439	-117.1887	2003	14139800	45.4446	-122.1095	unburned
11062001	34.2122	-117.4581	2003	14141500	45.4154	-122.1715	unburned
11113500	34.3956	-119.0765	2017	14154500	43.736	-122.8734	unburned
11124500	34.5967	-119.9088	2007	14158500	44.361	-121.9956	2003
11132500	34.5886	-120.4085	unburned	14158790	44.3346	-122.047	unburned
11143000	36.2458	-121.7733	2008	14165000	44.0929	-122.9573	unburned
11152000	36.2805	-121.3227	2008	14178000	44.7068	-122.1012	2003
11176400	37.5613	-121.6838	2003	14182500	44.7915	-122.579	unburned
11186000	35.9452	-118.4776	2002	14185000	44.3918	-122.4976	unburned
11200800	35.9416	-118.8229	unburned	14185900	44.5401	-122.4359	unburned
11230500	37.3394	-118.9735	unburned	14226500	46.6129	-121.6793	unburned
11315000	38.5191	-120.2127	unburned	14236200	46.5954	-122.4596	unburned
11316800	38.4032	-120.4469	unburned	14305500	44.7151	-123.8873	unburned
11367500	41.1882	-122.0656	unburned	14308000	42.9304	-122.9484	2002
11379500	40.0246	-122.5097	unburned	14309500	42.804	-123.6109	unburned
11381500	40.0546	-122.0242	1999	14316700	43.3498	-122.7289	unburned
11383500	40.014	-121.9483	1990	14325000	42.8915	-124.0707	unburned
11427700	39.1357	-120.4785	2001	14332000	42.7082	-122.3928	unburned
11449500	38.9274	-122.8436	2015	14400000	42.1234	-124.1873	2017
11451100	39.1654	-122.62	2018				

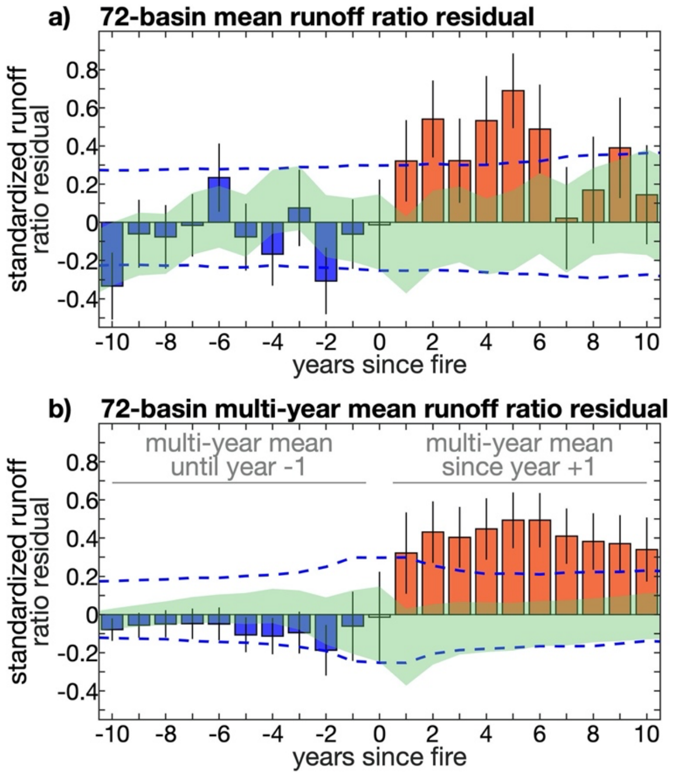

Supplementary Figure S1. Effect of forest fire on water-year runoff ratio. (a) Average standardized runoff ratio residual among burned basins in years prior to (blue bars), during (clear bar), and after (orange bars) each basin's largest forest fire year. (b) Same as (a) but for multi-year means leading up to and following each basin's forest-fire year (e.g., year 6 is the post-fire 6-year mean). For this analysis, a time series of runoff ratio residuals was calculated for each basin by first calculating a time series of runoff ratio residuals (observed minus modeled) and then standardizing relative to pre-fire years. Black vertical lines: 90% confidence intervals in multi-basin means. Blue dashed lines: Inner 90% of 10,000 repetitions when residuals are replaced with random time series with pre-fire variance and autocorrelation. Green area: Inner 90% of 10,000 repetitions when burned basins are replaced with random unburned basins.

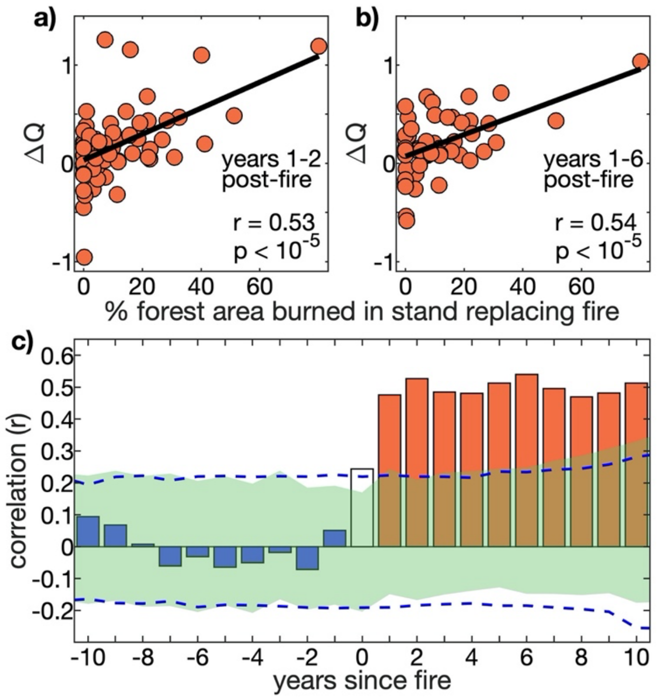

Supplementary Figure S2. Severity of forest fire affects post-fire runoff boost. (a and b) Inter-basin regression of the streamflow offset (ΔQ) in the first 2 and 6 years postfire, respectively, against the percent of forest area that was burned by stand-replacing fire according to the dataset developed by Parks and Abatzoglou (4). (c) Inter-basin correlation when repeating the analyses from (a and b) for other multi-year periods leading up to (blue bars) and after (orange bars) the fire year (clear bar). In (c), blue dashed lines bound inner-90% range when repeating 10,000 times with random time series with pre-fire variance and autocorrelation, green area bounds inner-90% range when repeating 10,000 times with random unburned basins. In this analysis there are 64 sample points, compared to 72 in our primary analyses, because the Parks and Abatzoglou dataset does not have data for all fires in the current MTBS database. In (a and b), years 1–2 and years 1–6 are shown because years 1–2 are when the all-basin mean streamflow enhancement is first significantly positive (Fig. 1e) and years 1–6 represent the full post-fire period when all-basin mean streamflow was positive in all years (Fig. 1d).

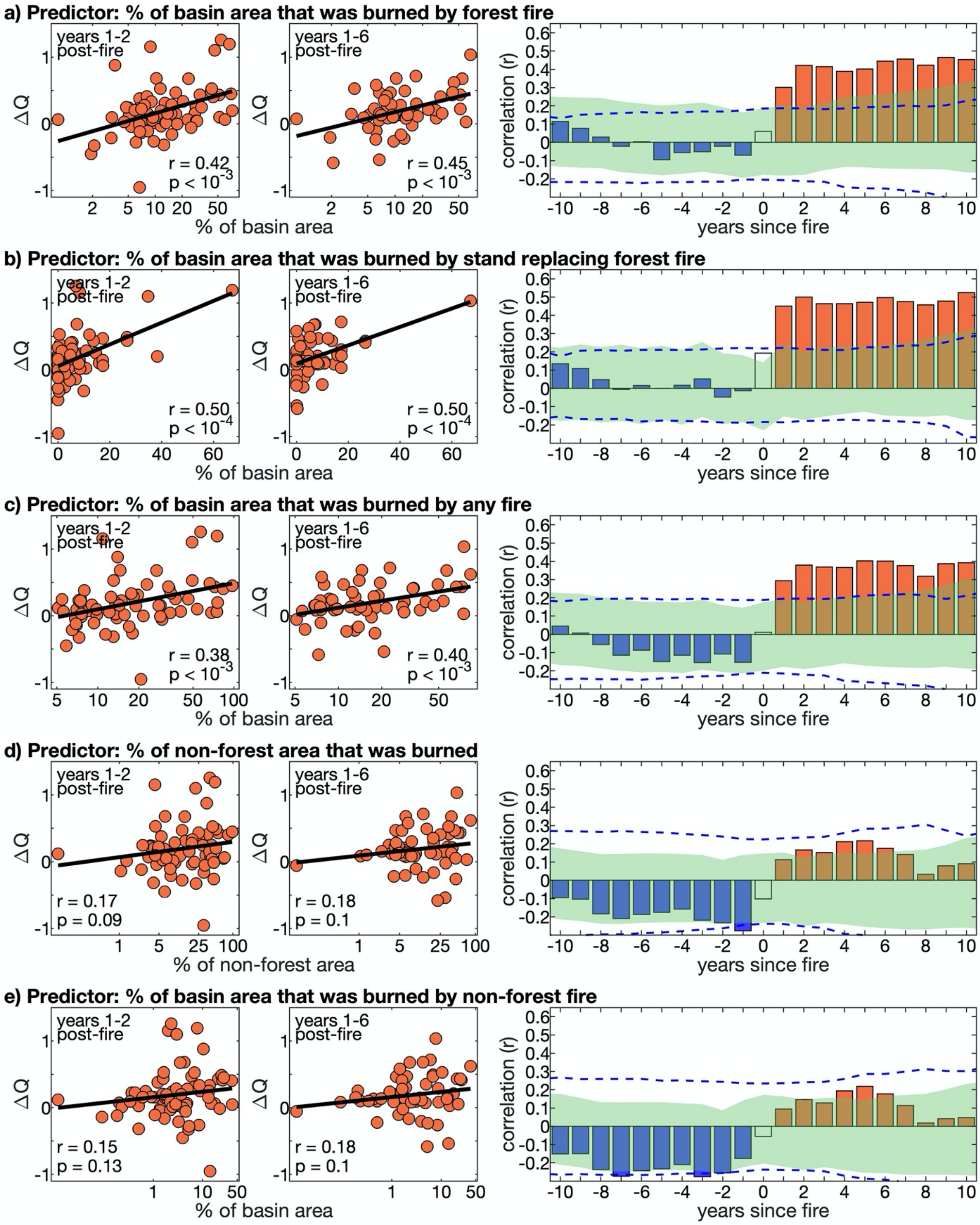

Figure S3. Correlation between post-fire runoff changes and various metrics representing burned area or severity. In each row, the three panels are alternate versions of the three panels in Figure 2 and Supplementary Figure S2. Scatter plots show inter-basin regressions of the streamflow offset (ΔQ) averaged over the first (left) 2 and (right) 6 years post-fire, respectively, against (a) the percentage of each basin's total area that burned in forest fire during the fire year, (b) the percentage of each basin's total area that burned in stand-replacing forest fire during the fire year, (c) the percentage of each basin's total area that burned in any wildfire (across any vegetation type and

of any severity), (d) the percentage of each basin's non-forest area that burned in non-forest area, and (e) the percentage of each basin's total area that burned in non-forest fire. Bar plots show correlations between each row's predictor variable and (ΔQ) averaged across multi-year periods leading up to (blue bars) and after (orange bars) the fire year (clear bar). Blue dashed lines: Inner 90% when each basin's ΔQ is replaced with 10,000 synthetic time series with pre-fire variance. Green area: Inner 90% of 10,000 repetitions with unburned basins. In scatter plots, years 1–2 and years 1–6 are shown because years 1–2 are when the all-basin mean streamflow enhancement is first significantly positive (Fig. 1e) and years 1–6 represent the full post-fire period when all-basin mean streamflow was positive in all years (Fig. 1d).

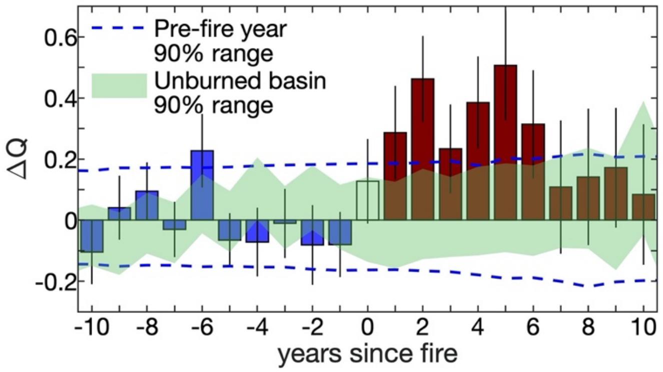

Supplementary Figure S4. Mean water-year streamflow offsets before and after fire among heavily burned basins. (a–e) Streamflow offsets (ΔQ) for (a) the water year, averaged across only the 40% of burned basins where >20% of forest area burned in the fire year. Colored bars: multi-year means leading up to (blue) and after (dark red) the fire year. Clear bar: fire year. Black vertical lines: 90% confidence intervals in multi-basin means. Blue dashed lines: Inner 90% of 10,000 repetitions with random time series with pre-fire variance and autocorrelation. Green background: Inner 90% of 10,000 repetitions with random unburned basins.

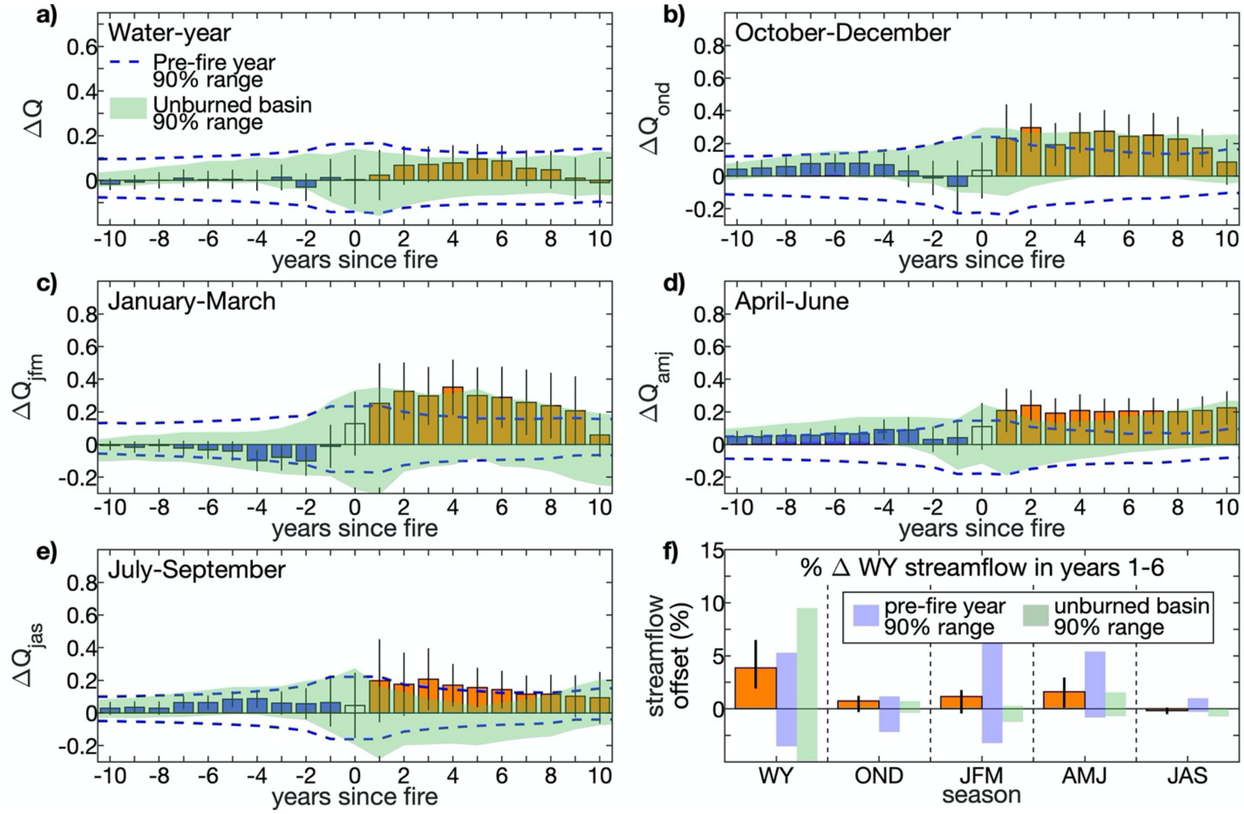

Supplementary Figure S5. Multi-year mean streamflow offsets before and after fire in lightly burned basins. (a–e) Streamflow offsets (ΔQ) for (a) the water year and (b–e) each of the four seasons, averaged across only basins where <20% of forest area burned in the fire year. Colored bars: multi-year means leading up to (blue) and after (orange) the fire year. Clear bar: fire year. Black vertical lines: 90% confidence intervals in multi-basin means. (f) Orange red bars: Multi-basin median streamflow offset averaged across years 1–6 post-fire, expressed as percent of estimated total water-year streamflow for the water year (WY) and seasons October–December (OND), January–March (JFM), April–June (AMJ), and July–September (JAS). Black vertical lines: 90% confidence intervals in (a–e) mean and (f) median values. Blue dashed lines in (a–e) and blue vertical areas in (f): Inner 90% of 10,000 repetitions with random time series with pre-fire variance and autocorrelation. Green background in (a–e) and green vertical areas in (f): Inner 90% of 10,000 repetitions with random unburned basins.

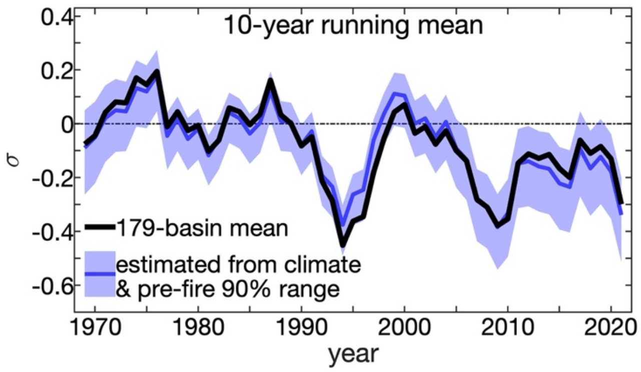

Supplementary Figure S6. Observed streamflow versus expectations from climate. 10-year running mean of observed (black) and estimated (blue) standardized water-year streamflow (Q) anomalies (σ) averaged across all 179 forested basins considered in this study. For the 72 burned basins, streamflow is estimated based on pre-fire fire-climate relationships. Blue area and line: Inner 90% and median of 10,000 repetitions with random time series with pre-fire variance and autocorrelation.

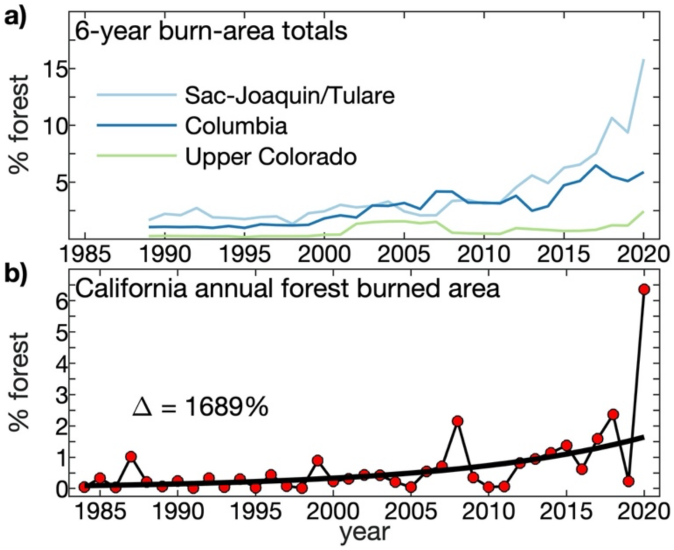

Supplementary Figure S7. Time series of forest-fire area, 1984–2020. (a) Six-year running total percent of forest area burned in three major watersheds. Line colors correspond to the three large watersheds outlined in Figure 5d. (b) Annual percent of forest burned in California (trend line fit by applying the Theil Sen linear trend estimator to the logarithm of burned area, delta value indicates relative change in burned area according to the trend line).

Supplementary References

1. J. Eidenshink, *et al.*, A project for monitoring trends in burn severity. *Fire Ecol* **3**, 3–21 (2007).

2. K. C. Short, Sources and implications of bias and uncertainty in a century of US wildfire activity data. *Int J Wildland Fire* **24**, 883–891 (2015).

3. L. Giglio, W. Schroeder, C. O. Justice, The collection 6 MODIS active fire detection algorithm and fire products. *Remote Sens Environ* **178**, 31–41 (2016).

4. S. A. Parks, J. T. Abatzoglou, Warmer and drier fire seasons contribute to increases in area burned at high severity in western US forests from 1985 to 2017. *Geophys Res Lett* **47**, e2020GL089858 (2020).

5. A. P. Williams, *et al.*, Large contribution from anthropogenic warming to a developing North American megadrought. *Science* **368**, 314–318 (2020).

6. R. S. Vose, *et al.*, Improved historical temperature and precipitation time series for US climate divisions. *J Appl Meteorol Clim* **53**, 1232–1251 (2014).

7. J. W. Oyler, A. Ballantyne, K. Jencso, M. Sweet, S. W. Running, Creating a topoclimatic daily air temperature dataset for the conterminous United States using homogenized station data and remotely sensed land skin temperature. *Int J Climatol* **35**, 2258–2279 (2015).

8. C. Daly, *et al.*, Physiographically sensitive mapping of climatological temperature and precipitation across the conterminous United States. *Int J Climatol* **28**, 2031–2064 (2008).

9. Y. Xia, *et al.*, Continental-scale water and energy flux analysis and validation for the North American Land Data Assimilation System project phase 2 (NLDAS-2): 1. Intercomparison and application of model products. *J Geophys Res-Atmos* **117**, D03109 (2012).

10. J. Sheffield, G. Goteti, E. F. Wood, Development of a 50-yr high-resolution global dataset of meteorological forcings for land surface modeling. *J Climate* **19**, 3088–3111 (2006).

11. J. L. Monteith, Evaporation and Environment. *Sym Soc Exp Biol* **19**, 205–234 (1965).

12. R. G. Allen, L. S. Pereira, D. Raes, M. Smith, "Crop evapotranspiration-Guidelines for computing crop water requirements-FAO Irrigation and drainage, paper 56" (Food and Agriculture Organization of the United Nations, 1998).

13. G. D. Farquhar, Carbon dioxide and vegetation. *Science* **278**, 1411 (1997).

14. Y. Yang, M. L. Roderick, S. Zhang, T. R. McVicar, R. J. Donohue, Hydrologic implications of vegetation response to elevated CO2 in climate projections. *Nat Clim Change* **9**, 44–48 (2019).

15. J. T. Abatzoglou, Development of gridded surface meteorological data for ecological applications and modelling. *Int J Climatol* **33**, 121–131 (2013).

16. S. S. Borkotoky, A. P. Williams, S. Steinschneider, E. R. Cook, Reconstructing extreme precipitation in the Sacramento River watershed using tree-ring based proxies of cold-season precipitation. *Water Resour Res* **57**, e2020WR028824 (2021).

17. D. A. Hastings, P. K. Dunbar, "Global Land One-kilometer Base Elevation (GLOBE) Digital Elevation Model, Documentation, Volume 1.0" (National Oceanic and Atmospheric Administration, National Geophysical Data Center, 1999).
